# A Case of Carotid Sinus Reflex Caused by Manual Aspiration Thrombectomy Using a Balloon Guide Catheter

**DOI:** 10.7759/cureus.56253

**Published:** 2024-03-16

**Authors:** Yuki Kubota, Fusakazu Oya, Fumiko Higashiyama

**Affiliations:** 1 Neurosurgery, Shinshu Ueda Medical Center, Ueda, JPN

**Keywords:** carotid sinus reflex, manual aspiration thrombectomy, cardiogenic cerebral embolism, balloon guide catheter, acute ischemic stroke (ais)

## Abstract

When starting a mechanical thrombectomy, manual aspiration with balloon guide catheters inserted into the internal carotid artery (ICA) is an efficient method for thrombus aspiration. However, no complications associated with this procedure have been reported. This study describes the case of a 76-year-old man who presented to our hospital with total aphasia and complete right-sided paralysis due to chronic atrial fibrillation and left occlusion of the ICA. When the balloon guide catheter was inserted and inflated at the origin of the left ICA, the patient’s systolic blood pressure suddenly decreased from 114 mm Hg to 44 mm Hg. This sudden hypotension may have been caused by the carotid sinus reflex. Hypotension improved following balloon deflation. The procedure was continued, resulting in complete recanalization of the left ICA. The patient died from acute exacerbation of interstitial pneumonia. Although this complication is rare, similar phenomena have been recognized in carotid artery stenting and the use of flow-diverting devices. To the best of our knowledge, this is the first report of a case wherein the carotid sinus reflex was induced by manual aspiration using a balloon guide catheter placed in the ICA. Clinicians should recognize the importance of ensuring that the proximal end of the balloon crosses the carotid sinus when dilating and occluding the ICA with a balloon to avoid the carotid sinus reflex.

## Introduction

Manual aspiration using balloon guide catheters placed in the internal carotid artery (ICA) is effective for thrombus aspiration before mechanical thrombectomy [1-3]. Particularly, this technique is efficient for managing potentially significant blood clots in the ICA in patients with cardiogenic cerebral embolism [1].

The carotid sinus reflex regulates arterial blood pressure by causing bradycardia and hypotension [4]. The carotid sinus reflex is activated during carotid artery stenting and the use of flow-diverting devices during neuroendovascular therapy [5-9]. This activation has been attributed to mechanical compression by the balloon, stent, or catheter during the procedure.

To the best of our knowledge, no studies have reported complications during mechanical thrombectomy via manual aspiration using balloon guide catheters placed in the ICA. In this study, we present a case of ischemic stroke due to ICA occlusion where manual aspiration using balloon guide catheters in the ICA caused the carotid sinus reflex.

## Case presentation

**Figure 1:**
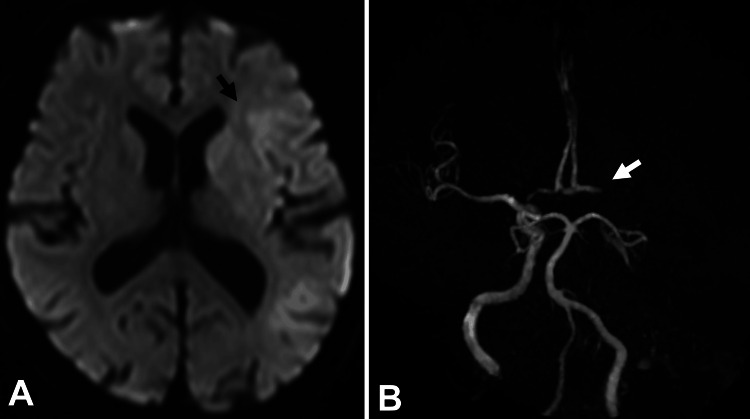
Preprocedural MRI and MRA. A. A diffusion-weighted MRI shows a faint high-intensity lesion in the area of the left ICA (black arrow). B. Cranial MRA revealing occlusion in the left ICA (white arrow). MRI, magnetic resonance imaging; MRA, magnetic resonance angiogram; ICA, internal carotid artery

A 76-year-old male patient with chronic atrial fibrillation who had not been taking anticoagulants experienced sudden total aphasia and complete right-sided paralysis. Neurological evaluation revealed a National Institutes of Health Stroke Scale score of 20. Diffusion-weighted magnetic resonance imaging (DW-MRI) revealed high-intensity areas in the left frontal, parietal, and temporal brain lobes (Figure 1A). Moreover, the left ICA could not be visualized on magnetic resonance angiography (Figure 1B). The patient was administered 0.6 mg/kg alteplase and subsequently transferred to the angiography room for mechanical thrombectomy.

**Figure 2:**
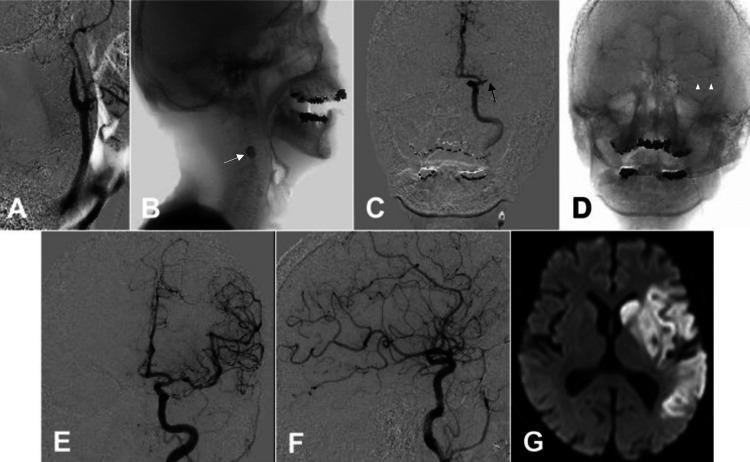
Intraprocedural cervical and cranial angiogram and postprocedural magnetic resonance imaging (MRI). A. A lateral view of the left common carotid angiogram showing occlusion of the origin of the left ICA. B. Inflation of the balloon of the Optimo 8 Fr (Tokai Medical Products, Aichi, Japan) (white arrow) was followed by a decrease in systolic blood pressure. C. An anteroposterior view of the left internal carotid angiogram showing occlusion in the left MCA (black arrow). D. An Embotrap III 5 × 37 mm (Cerenovus, Fremont, CA, USA) (white arrowheads) was used. E, F. Angiograms showing complete recanalization of the left ICA and intracranial arteries, with TICI score of 3; E. Cranial anteroposterior view, F. Cranial lateral view. G. Diffusion-weighted MRI showing cerebral infarction in the area of the left ICA. ICA, internal carotid artery; MCA, middle cerebral artery; TICI, thrombolysis in cerebral infarction

In the right femoral artery, an 8.2 Fr sheath (Medikit, Tokyo, Japan) was inserted. An Optimo 8 Fr balloon guide catheter (Tokai Medical Products, Aichi, Japan) was inserted into the left common carotid artery using the coaxial method with a 5 Fr modified Simmons catheter (Hanaco Medical, Saitama, Japan) and a 0.035-inch SURF guidewire (Piolax Medical Devices Inc., Kanagawa, Japan). A left common carotid angiography showed that the left ICA was occluded (Figure 2A). After positioning the Optimo 8 Fr balloon guide catheter at the origin of the left ICA, the balloon was inflated to block blood flow within the ICA (Figure 2B). Suddenly, the patient’s systolic blood pressure (sBP) decreased from 114 mm Hg to 44 mm Hg. Methylprednisolone (125 mg) and dopamine (5 mL/h) intravenous injections were administered owing to suspected anaphylactic shock caused by the contrast medium. Manual aspiration was conducted using a 20-mL syringe connected to the primary port of the Optimo 8 Fr balloon guide catheter. A large volume of dark-red thrombi was aspirated. After deflating the balloon, sBP returned to 90 mm Hg. Consequently, repeat left internal carotid angiography showed that the left middle cerebral artery (MCA) was occluded (Figure 2C).

A Phenom 27 microcatheter (Medtronic, Minneapolis, MN, USA) was advanced via a Penumbra RED 62 reperfusion catheter (Penumbra, Alameda, CA, USA) over a Synchro-Select Soft microwire (Stryker Neurovascular, Fremont, CA, USA) in the left MCA. The microcatheter was maneuvered over the microguidewire through the thrombus, and the aspiration catheter was positioned to make contact with the thrombus. An Embotrap III 5 × 37 mm (Cerenovus, Fremont, CA, USA) was deployed via the microcatheter distally to proximally across the occlusion site (Figure 2D). Next, the microcatheter was withdrawn to create a larger cross-sectional luminal area for aspiration. Subsequently, the Embotrap III and Penumbra RED 62 were removed as a single unit. Angiography revealed full recanalization of the left ICA and intracranial arteries, with a thrombolysis in cerebral infarction score of 3 (Figures 2E, 2F). The puncture-to-reperfusion time was 31 minutes. Puncture site hemostasis was achieved using manual compression. The patient’s blood pressure did not decrease again during endovascular therapy.

After endovascular therapy, the patient was closely monitored, and no decrease in heart rate or blood pressure was noted. The administration of intravenous dopamine was discontinued 2 hours postoperatively. The patient was diagnosed with cardiogenic cerebral embolism due to chronic atrial fibrillation and commenced on a daily dose of 30 mg of edoxaban starting the next day. Postoperative DW-MRI revealed a cerebral infarction in the left frontal lobe (Figure 2G).

Severe right-sided paralysis and total aphasia persisted. The patient died from acute exacerbation of interstitial pneumonia 35 days postoperatively. A pathological autopsy was not performed.

## Discussion

This case illustrates two important clinical issues. First, manual aspiration using balloon guide catheters in the ICA can induce the sinus reflex due to the balloon compressing the carotid sinus. Second, inflating the balloon at least 13 mm distal to the carotid bifurcation is essential for preventing the carotid sinus reflex during manual aspiration using balloon guide catheters.

Manual aspiration using balloon guide catheters placed in the ICA enables thrombus aspiration before thrombectomy [1-3]. This method is effective for handling potentially substantial thrombi in the proximal ICAs of patients with cardiogenic cerebral embolisms. This approach reduces the time from puncture to reperfusion by eliminating the need for extra steps during thrombus aspiration [3]. Obviating the need for additional steps, such as using a microcatheter or deploying a stent retriever, facilitates a less invasive thrombectomy through straightforward manual aspiration and enables a seamless transition to subsequent procedures, including deploying a stent retriever or using the direct aspiration first-pass technique [1]. Manual aspiration of the thrombus using balloon guide catheters in the common carotid artery is a feasible procedure [10]. However, during carotid artery stenting, the flow from the external carotid artery to the ICA is reversed after balloon occlusion of the common carotid artery [11]. Thus, this might lead to inadequate aspiration pressure in the ICA because of concurrent blood aspiration from the external carotid artery during manual aspiration from the common carotid artery [11].

The carotid sinus reflex regulates arterial blood pressure [12]. The baroreceptors accountable for this reflex are located in the carotid sinuses near the carotid bifurcation, and they react to the stretching of the arterial wall [12]. Specifically, if arterial pressure suddenly rises, the arterial walls expand passively, stimulating these receptors [12]. These signals are transmitted via the glossopharyngeal nerve to the nucleus tractus solitarii in the medulla [12]. Efferent fibers carry impulses through the sympathetic adrenergic and vagus nerves, influencing the sinus node of the heart, the atrioventricular node, and various blood vessels throughout the body [13]. The carotid sinus reflex is categorized into the following three types: (1) the cardioinhibitory type (70-75% of cases), characterized by bradycardia or asystole lasting more than 3 seconds; (2) the vasodepressor type (5-10% of cases), characterized by a decrease in sBP >50 mm Hg; and (3) the mixed type (20-25% of cases), characterized by a decrease in sBP >50 mm Hg and bradycardia [14]. Accordingly, we concluded that balloon inflation in the carotid sinus caused a vasodepressor-type carotid sinus reflex in our patient.

Activation of the carotid sinus reflex is reportedly a complication of carotid artery stenting and flow diverter deployment [5-9]. During these procedures, the carotid sinus reflex is thought to be induced by the mechanical stretching of the carotid sinus baroreceptor, either during balloon inflation, stent application, or catheter compression. While reflex activation induced by balloon inflation typically subsides swiftly, using a self-expanding stent tends to prolong this response [15].

Manual aspiration thrombectomy using a balloon guide catheter and balloon inflation in the carotid sinus can excessively stimulate carotid sinus baroreceptors. The mean length of the carotid sinus on the ICA is 9.99 ± 2.22 mm from the carotid bifurcation [16]. Furthermore, a positive correlation was found between sinus length and age, with higher values in older patients [16]. Therefore, it is essential to expand the balloon only after the proximal end of the balloon has been advanced at least 13 mm beyond the carotid artery bifurcation. In this case, the reflex was so strongly activated that it caused a transient decrease in sBP; however, it resolved quickly. This finding supported the hypothesis that activation of the carotid sinus reflex-induced hypotension.

This report has a few limitations. Although the contrast agent may have caused the drop in blood pressure, it is more plausible to attribute this to a reflex triggered by the balloon. This is evidenced by the decrease in blood pressure upon balloon inflation and subsequent improvement upon balloon deflation. Furthermore, it typically takes an average of 10 minutes for blood pressure to decrease following the administration of contrast agents [17]. In the present case, it took more than 10 minutes from administering the contrast agent to the onset of blood pressure reduction. In addition, despite using contrast agents after deflating the balloon, no decrease in blood pressure was observed. For these reasons, the effect was probably induced by balloon dilation in this case. Further studies are needed to fully understand the relationship between balloon inflation in the ICA and the carotid sinus reflex and to improve the management of the carotid sinus reflex in patients undergoing mechanical thrombectomy.

## Conclusions

In this study, we report a case of a rare but serious complication of manual aspiration using a balloon guide catheter. A balloon guide catheter was inserted into the origin of the ICA, followed by balloon expansion, which induced the carotid sinus reflex. Hence, if manual aspiration is attempted using an inflated balloon guide catheter, the catheter should be positioned distal to the carotid sinus. This case report describes one of the potential complications of manual aspiration thrombectomy using a balloon guide catheter.
